# Single-cell transcriptomics and Mendelian randomization reveal LUCAT1’s role in right-sided colorectal cancer risk

**DOI:** 10.3389/fgene.2024.1357704

**Published:** 2024-04-22

**Authors:** Zhihao Shang, Songyang Xi, Yueyang Lai, Haibo Cheng

**Affiliations:** ^1^ Jiangsu Collaborative Innovation Center of Traditional Chinese Medicine Prevention and Treatment of Tumor, Nanjing University of Chinese Medicine, Nanjing, China; ^2^ The First School of Clinical Medicine, Nanjing University of Chinese Medicine, Nanjing, China; ^3^ Zhenjiang Hospital of Chinese Traditional and Western Medicine, Zhenjiang, Jiangsu, China

**Keywords:** colorectal cancer, single-cell sequencing, Mendelian randomized, LUCAT1, bioinformatics

## Abstract

**Background:** Colorectal cancer (CRC) is a malignancy with high incidence and mortality rates globally, categorized into left-sided and right-sided CRC, each exhibiting significant differences in molecular characteristics, clinical manifestations, and prognosis.

**Methods:** This study employed single-cell transcriptomic data and various bioinformatics approaches, such as two-sample Mendelian randomization, reverse Mendelian randomization, colocalization analysis, directed filtering, pseudotime analysis, and intercellular communication analysis. It analyzed cellular-level disparities between left-sided and right-sided CRC, identifying distinct subpopulations with characteristic variations. For these cells, two-sample Mendelian randomization was utilized to explore gene-to-one-sided CRC causality.

**Results:** LUCAT1 was enriched in high-abundance monocyte subpopulations in right-sided CRC and demonstrated potential risk factor status through Mendelian randomization analysis. The specific single-nucleotide polymorphism (SNP) rs10774624 was associated with an increased risk of CRC. Moreover, metabolic pathway analysis revealed that LUCAT1^+^ monocytes exhibit lower communication activity in the tumor microenvironment and heightened activity in metabolic functions like glycosaminoglycan degradation. Its biological functions are related to the positive regulation of interleukin-6 production and NF-kappa B signaling, among others.

**Conclusion:** This study confirmed a potential causal relationship between LUCAT1 and right-sided CRC risk through Mendelian randomization analysis. These findings provide novel insights into the pathogenesis of right-sided CRC and may aid in developing early detection and treatment strategies for right-sided CRC.

## 1 Introduction

Colorectal cancer (CRC) ranks among the most prevalent and lethal malignancies worldwide (White and Sears, 2023). This neoplasm is categorized into right-sided and left-sided colorectal cancer based on the tumor’s location within the colon. This classification extends beyond mere anatomical delineation, as these subtypes exhibit significant disparities in molecular characteristics, clinical manifestations, treatment responses, and prognoses. Typically, right-sided CRC is more common in older patients and is associated with a poorer prognosis, whereas left-sided CRC often responds better to treatment and shows higher survival rates (Boeckx et al., 2017; Nawa et al., 2008). These differences underscore the need for personalized approaches in the diagnosis, treatment, and management of CRC, highlighting the importance of understanding these variations to optimize treatment strategies, improve patient outcomes, and develop novel therapeutic targets.

Molecularly, right-sided and left-sided CRC demonstrate distinct characteristics. Right-sided CRC is frequently linked with microsatellite instability (MSI) and BRAF mutations, associated with immune evasion and chemotherapy resistance (Takahashi et al., 2016; van der Post and Hansson, 2014; Weiss et al., 2011). Conversely, left-sided CRC often exhibits mutations in the KRAS and p53 genes, aligning with the typical adenoma-carcinoma sequence (Brooks et al., 2001). Additionally, right-sided CRC shows higher genomic and epigenetic heterogeneity, while left-sided CRC is characterized by chromosomal instability (Kajiwara et al., 2023). These molecular features not only influence the tumor’s biological behavior but also critically impact the response to various treatment modalities, playing a pivotal role in clinical decision-making. Thus, a deeper understanding of these molecular differences is crucial for developing more precise and effective treatment approaches.

Lung cancer-associated transcript 1 (LUCAT1), a long non-coding RNA (lncRNA), has garnered attention for its expression and function in various tumors. Initially identified in lung cancer, LUCAT1 has been found to regulate tumor progression in other cancer types as well (Cao et al., 2023). Its roles include promoting tumor cell proliferation, inhibiting apoptosis, enhancing cancer cell migration, and invasion, and participating in epigenetic regulation. LUCAT1 also modulates the activity of microRNAs (miRNAs) by acting as an “miRNA sponge,” indirectly influencing the expression of numerous genes. A more comprehensive understanding of LUCAT1’s role in tumorigenesis may pave the way for developing novel cancer treatment strategies (Xiao et al., 2021).

Single-cell technologies enable researchers to analyze gene expression, molecular characteristics, and cell states at an individual cell level. This is significant for revealing tumor heterogeneity, identifying distinct cell subpopulations, and understanding cell interactions within the tumor microenvironment. Mendelian randomization, an epidemiological method using genetic variants as instrumental variables, assesses causal relationships between exposures and disease outcomes. This approach helps mitigate confounding and reverse causation issues common in traditional observational studies (Lee et al., 2023; Shigemura et al., 2023; Wang H. et al., 2023; Wang M. et al., 2023).

Our study, leveraging single-cell transcriptomic data from right-sided and left-sided CRC obtained from the Gene Expression Omnibus (GEO) database, identifies a subgroup of mononuclear cells that promote tumor development. Through analyzing expression differences with other cell subpopulations, we identified differentially expressed genes in this subgroup. Using these genes as exposure factors and employing bioinformatics methods like Mendelian randomization, colocalization, and directional filtering, we discovered that genetic variations in LUCAT1, a long-chain non-coding RNA, are risk factors for right-sided CRC, potentially linked to the single-nucleotide polymorphism (SNP) rs10774624. These findings offer insights into the molecular mechanisms of right-sided and left-sided CRC and contribute to developing potential targeted therapeutic strategies.

## 2 Materials and methods

### 2.1 Data acquisition and preprocessing

The data for this study were sourced from the publicly accessible GEO database, specifically the GSE188711 dataset, which comprises single-cell RNA sequencing data from six colorectal cancer samples, including three from the left side and three from the right side of the colon. We used the Read10X function from the Seurat package to import data in the 10x Genomics format for data preprocessing. Each sample’s data were read from the specified directory and immediately encapsulated into a Seurat object using the CreateSeuratObject function, with parameters set to default except for the project argument, which was uniquely assigned to each sample based on its origin (e.g., L1, L2, and L3 for left-sided samples and R1, R2, and R3 for right-sided samples).

### 2.2 Quality control and dimension reduction

The Seurat objects corresponding to individual samples were merged into a single dataset using the merge function with the default parameters to facilitate collective analysis. During the quality control (QC) phase, metrics such as the number of gene expression features (nFeature_RNA), the proportion of mitochondrial gene expression (percent.mt), and the proportion of hemoglobin gene expression (percent.HB) were computed for each cell. Cells were then filtered based on the following criteria: cells with gene counts over 200 and under 4,000 and mitochondrial gene expression below 10% to eliminate both dead or dying cells and potential doublets or multiplets.

For dimensionality reduction, we utilized principal component analysis (PCA) using the RunPCA function with features = VariableFeatures (object) to focus on highly variable genes, followed by uniform manifold approximation and projection (UMAP) for visualization purposes, employing the RunUMAP function with the default parameters except for dims = 1:10 to use the first ten principal components (Qi and Zhang, 2023).

### 2.3 Clustering analysis and single-cell type annotation

Clustering analysis was performed using the FindNeighbors function with default parameters but specifying dims = 1:10 to use the data from the first ten principal components for neighborhood calculation. The FindClusters function was then applied for actual clustering, with the resolution parameter adjusted based on preliminary analyses to optimize cluster granularity. We utilized the SingleR package or cell-type annotation, leveraging the SingleR function with the reference dataset obtained from the HumanPrimaryCellAtlasData function for human samples. Annotation was performed by matching our dataset’s expression profiles with those in the reference, providing a predicted cell type for each cell. In particular, for the detailed analysis of monocyte subpopulations, we isolated monocyte cells and conducted further dimension reduction, clustering, and annotation steps. The FindVariableFeatures function was used to select 2000 highly variable genes, followed by PCA and UMAP for visualization. Harmony was used for batch correction using the RunHarmony function with group.by.vars = “orig.ident,” ensuring the integration of data from different samples without batch effects. The FindAllMarkers function was employed to identify subgroup-specific marker genes, with the parameters set to only.pos = TRUE, min.pct = 0.25, and logfc.threshold = 0.25 to focus on genes that were positively expressed in at least 25% of cells within any given cluster with a minimum fold change of 0.25.

This detailed methodology ensures a comprehensive and reproducible approach to analyzing single-cell RNA sequencing data, facilitating the identification of cellular subpopulations and their respective marker genes within the complex landscape of colorectal cancer (Feng et al., 2023).

### 2.4 Pseudotime and intercellular communication analysis

Leveraging the slingshot package, we conducted trajectory analysis on cells to delineate the developmental pathways of monocyte subtypes. Constructing SingleCellExperiment objects allowed us to convert Seurat objects for analysis with slingshot. Within the scope of the slingshot analysis, we designated “celltype” as the clustering label and employed UMAP for dimensionality reduction. Specific starting clusters [start.clus = c (3,5)] were selected, alongside setting the trajectory shrinkage parameter (shrink = 0.2) to facilitate the refined formation of trajectories. Additionally, trajectory visualization was performed, utilizing color and layout options provided by the RColorBrewer and igraph packages, graphically depicting cell state transitions and developmental trajectories.

In exploring intercellular communication, we initially refined our scRNA-seq dataset through a further filtration process, excluding non-monocyte cell types to concentrate on their communication within the colorectal cancer context. Subsequently, we engaged in a quantitative analysis of cell-to-cell communication using the CellChat package. This step involved the creation of a CellChat object and its integration with the Human Cell Communication Database (CellChatDB.human). Our focus was directed toward the “Secreted Signaling” category of cell communication, selecting supported ligand–receptor pairs from the database. By identifying overexpressed genes and ligand–receptor pairs and projecting these elements onto the PPI network, we could construct and quantify the probabilities of intercellular communication. Furthermore, we filtered the cell communication network, eliminating communications within specific cell groups that had fewer cells, adopting min.cells = 10 as a threshold for analysis. Finally, we depicted the network and bubble plots of cell communication through visualization tools, offering an intuitive presentation of the quantity and patterns of interactions among cell groups.

### 2.5 Metabolic pathway analysis

We employed the scMetabolism package to assess the metabolic activity within specific subpopulations of colorectal cancer cells. Initially, our focus was directed toward the “monocyte_CO2” subpopulation, which was further stratified into “LUCAT1^+^M” and “LUCAT1^−^M” subgroups based on the expression of the LUCAT1 gene. Additionally, subpopulations other than “monocyte_CO2” were isolated from the aggregate dataset for subsequent analysis. The scMetabolism package facilitated a quantitative assessment of metabolic activity in these cell subgroups. The specific steps of analysis included the following:

1) Utilizing the sc.metabolism.Seurat function to score metabolic pathways via the AUCell method, without imputation (imputation = F), and setting the number of parallel cores to 2 (ncores = 2). “KEGG” was selected as the metabolism type (metabolism.type = “KEGG”).
2) Certain metabolic pathways (for instance, input.pathway < - rownames (scRNA_metab @assays [["METABOLISM"]][["score"]])[61:90]) were chosen for dot plot visualization to illustrate the variability in pathway activity across different genotypes. Moreover, differential gene expression analysis was conducted using the FindAllMarkers function, identifying genes with significant expression differences between the “LUCAT1^+^M” and “LUCAT1^−^IM” subgroups (employing positive markers, with a log fold change threshold set at 0.5). The list of differential genes obtained was then utilized for subsequent functional enrichment analysis.

### 2.6 Gene conversion and Mendelian randomization preparation

Upon identifying key genes, we used the clusterProfiler and org.Hs.eg.db packages to convert these gene symbols to ENSEMBL IDs. This facilitated subsequent Mendelian randomization analysis, allowing us to extract SNP information related to these genes from publicly available Genome-Wide Association Studies (GWAS databases), serving as instrumental variables for the analysis.

### 2.7 Two-sample Mendelian randomization and bidirectional Mendelian randomization

In this study, differential genes in the second group of monocytes compared to other cells were used as exposure factors (Table 1), with colorectal cancer (CRC) as the outcome, for causal inference analysis (MR) using “TwoSampleMR” (https://github.com/MRCIEU/TwoSampleMR). The Wald ratio was used for genes with only one eQTL available, while inverse-variance weighted MR (MR-IVW) was applied when two or more genetic tools were available. The odds ratio (OR) of increased colorectal cancer risk was expressed for carriers of specific gene polymorphisms (e.g., a particular SNP) compared to non-carriers. For the primary analysis, the Bonferroni correction was applied for multiple testing adjustments, prioritizing results for further analysis with a threshold of 0.05/888 (*p* < 5.63 × 10^−5^). Moreover, colorectal cancer was also used as the exposure variable, with high expression of LUCAT1 in right-sided colorectal cancer monocytes as the outcome, for a bidirectional Mendelian randomization analysis, revealing potential bidirectional causal relationships between colorectal cancer onset and genes. Two colorectal cancer datasets from the GWAS database were selected, one as a test set (ebi-a-GCST90018808) and another as a validation set (ebi-a-GCST012879).

**TABLE 1:** Results of Mendelian randomization and colocalization with directional filtering.

Symbol	SNP	Mendelian randomization (Wald ratio)	Steiger filtering	Colocalization PPH4 (coloc.abf/coloc.susie)
LUCAT1	rs10774624	2.8365 (1.6750,4.8035)	Passed (3.20151 × 10^−5^)	9.297435e−01/0.00025

### 2.8 Colocalization analysis

Bayesian colocalization analysis was employed to assess the likelihood of two traits sharing the same causal variant, using the “Coloc” package (https://github.com/chr1sw) with default settings. As previously described, Bayesian colocalization provides posterior probabilities for five hypotheses regarding whether two traits share a single variant. In this study, we tested hypothesis 4 (PPH4), where LUCAT1 and colorectal cancer were associated with the region through a shared variant. Using the Coloc.abf and Coloc.susie algorithms, a gene was defined as having evidence of colocalization if it met the criterion of a gene-based PPH4 > 80% with at least one algorithm. Following the extraction and organization of relevant SNP data, the locusComparer package was used to create regional association plots, showcasing the association degree of specific gene regions with the occurrence of colorectal cancer. The specific steps of analysis included the following:

1) Employing the vcfR::read.vcfR function to read the “./eqtl-a-ENSG00000119917.vcf” file, obtaining eQTL information for the gene of interest.
2) Utilizing the separate function from the tidyverse package to process genotype data, splitting columns containing multiple pieces of information into separate variables, including effect size (beta), standard error (se), logarithmic *p*-value (logpvalue), allele frequency (eaf), and sample size (samplesize).
3) Calculating the minor allele frequency (MAF) and filtering data based on chromosomal position to focus on SNPs within specific regions of the gene.
4) Generating regional association plots with the locuscomparer package to visually depict the degree of association between the LUCAT1 gene eQTLs and the colorectal cancer GWAS results.

### 2.9 Directional filtering

Directional filtering analysis was conducted to evaluate the relative impact of SNPs on LUCAT1 gene expression and colorectal cancer occurrence. This step was achieved by comparing the association strength of SNPs with the exposure and outcome, aiming to ensure that the SNPs used in the analysis were correctly aligned on the causal pathway with the exposure variable. The steiger_filtering and directionality_test functions within the TwoSampleMR package were utilized for this analysis.

### 2.10 Gene ontology enrichment analysis and Kyoto Encyclopedia of Genes and Genomes pathway enrichment analysis

Gene ontology (GO) enrichment analysis and Kyoto Encyclopedia of Genes and Genomes (KEGG) pathway enrichment analysis are pivotal methodologies in bioinformatics for deciphering the biological processes and pathways underlying gene expression data. To investigate the potential biological functions of monocyte_CO2 cells and LUCAT1^+^ monocytes, we selected the top 100 highly variable genes from these two cellular subpopulations for GO and KEGG enrichment analyses. The org.Hs.eg.db package was utilized for ID conversion, while the clusterProfiler package facilitated the enrichment analyses. All analyses and visualizations were conducted within the R version 4.2.1 environment.

## 3 Results

### 3.1 Study design

**FIGURE 1:**
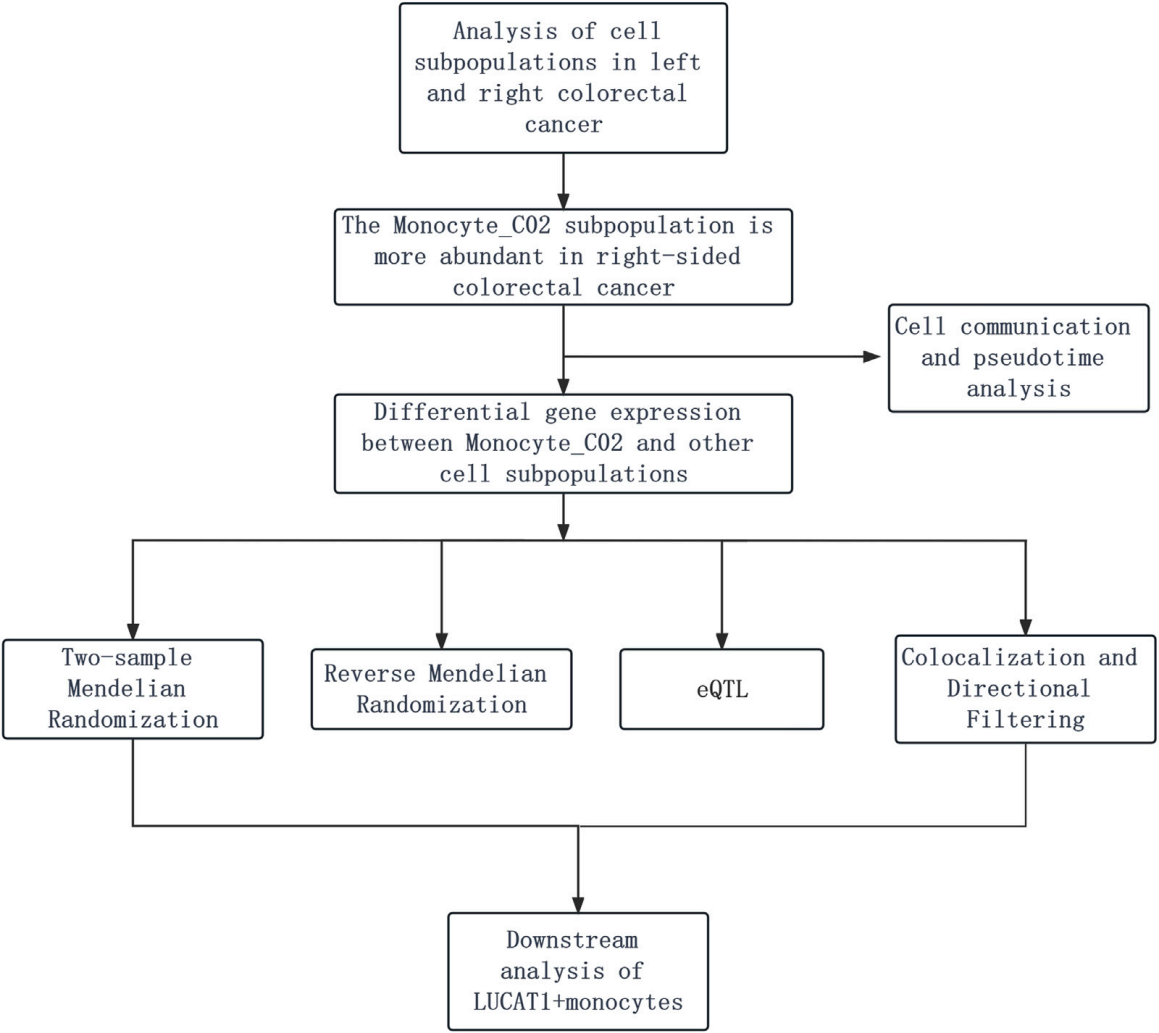
Depicts the analysis of colorectal cancer cell subpopulations, revealing a larger number of monocyte_CO2 cells in right-sided cases. It is followed by gene expression and cell interaction studies using Mendelian randomization and eQTL mapping, refined by colocalization and filtering, and ending with a focus on the role of LUCAT1^+^ monocytes in cancer progression.

Our investigation begins with the analysis of cell subpopulations in left- and right-sided colorectal cancer, identifying a notable monocyte_CO2 subpopulation predominance in right-sided cases. This observation is followed by differential gene expression analysis, coupled with cell communication and pseudotime analysis, to delineate gene expression patterns and cellular interactions. We then integrate two-sample Mendelian randomization, reverse Mendelian randomization, and eQTL mapping, refined by colocalization and directional filtering techniques. The study culminates with a focused downstream analysis of LUCAT1^+^ monocytes to elucidate their potential role in colorectal cancer progression (Figure 1).

### 3.2 Monocytes, particularly monocyte_CO2, exhibit greater abundancein right-sided colorectal cancer, potentially related to the characteristics of right-sided colorectal cancer

**FIGURE 2:**
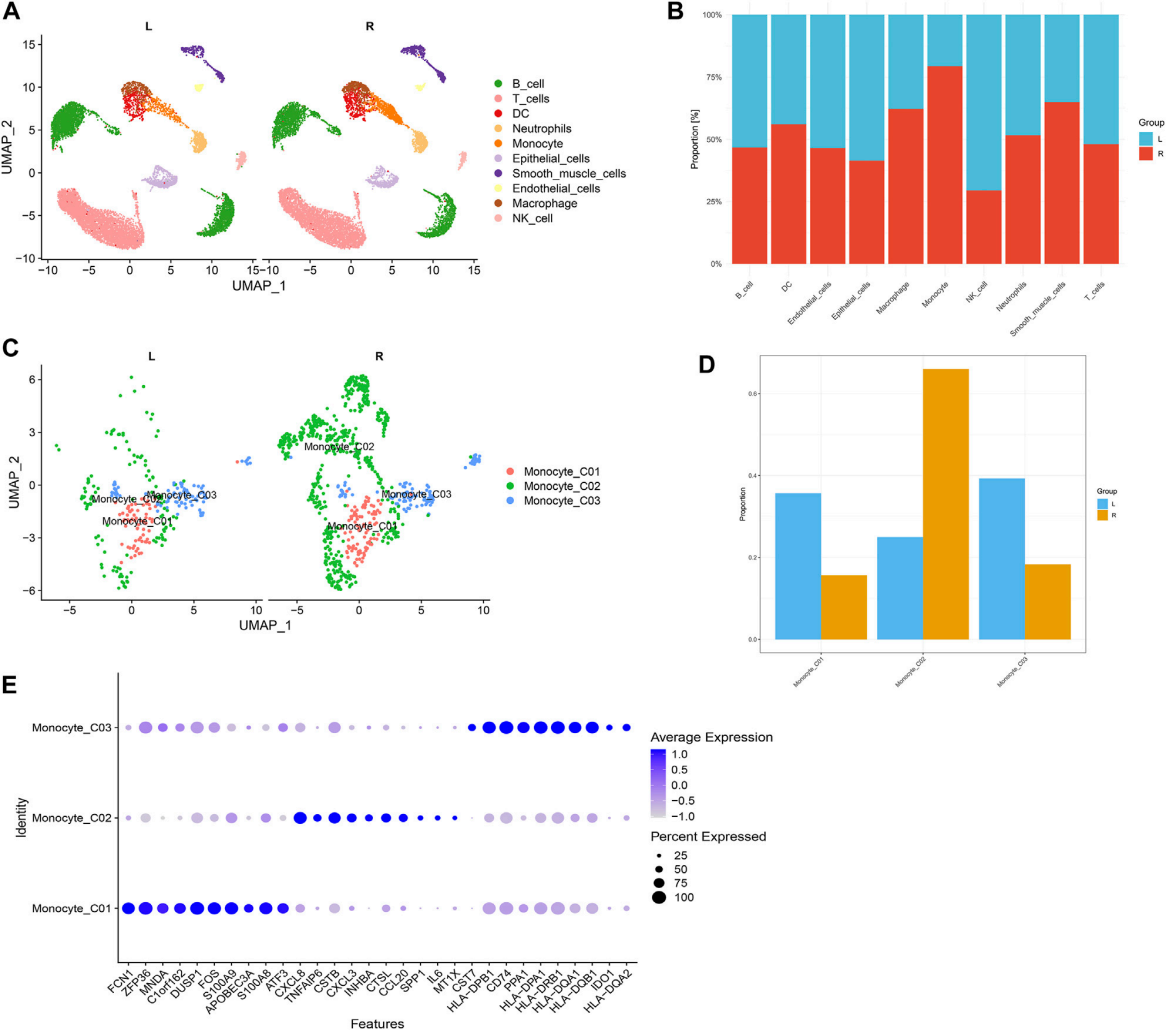
Single-cell transcriptomics reveal cellular disparities in left- and right-sided colorectal cancers. **(A)** Uniform manifold approximation and projection (UMAP) dimensionality reduction clustering identifies multiple cell subpopulations within colorectal cancer tissues, including B cells, T cells, dendritic cells, endothelial cells, epithelial cells, monocytes, neutrophils, smooth muscle cells, macrophages, and natural killer cells. **(B)** A stacked bar chart illustrates the comparative frequency of occurrence of each cell subpopulation between the two groups. **(C)** UMAP dimensionality reduction clustering displays the distribution of monocyte subpopulations. **(D)** Comparative cell proportion plots highlight the variance in monocyte subpopulations between left- and right-sided colorectal cancers. **(E)** Dotplot diagrams demonstrate the highly variable genes among three monocyte groups.

In this investigation, single-cell sequencing data from left- and right-sided colorectal cancer were retrieved from the GEO database, identified as dataset GSE188711. Following stringent quality control (Supplementary Figures S1A, B) and batch-effect normalization (Supplementary Figures S1C–E), we procured high-quality single-cell transcriptomic data. We initially performed dimensionality reduction clustering on individual samples, followed by the presentation of marker expression for each cell cluster post-merging (Supplementary Figures S2, S3). Utilizing UMAP for clustering analysis, we segregated cells within tumor tissues into multiple subgroups, including B cells, T cells, dendritic cells (DCs), endothelial cells, epithelial cells, monocytes, neutrophils, smooth muscle cells, macrophages, and natural killer (NK) cells, each displaying distinct distribution patterns in left- and right-sided colorectal cancer samples (Figure 2A). Cell ratio diagrams further disclosed a notably higher proportion of monocytes in right-sided than left-sided colorectal cancer (Figure 2B). An independent analysis of monocytes indicated a significantly elevated proportion of the monocyte_CO2 subpopulation in right-sided colorectal cancer, signifying that this distinct subpopulation might play an enhanced role in this cancer variant (Figures 2C, D).

Further investigation into these monocyte subgroups revealed expression patterns of highly variable genes (Figure 2E). A suite of such genes, including CXCL8, TNFAIP6, CXCL3, and SPP1, were specifically upregulated in the monocyte_CO2 cells. The expression patterns of these genes might correlate with the functional dynamics of monocytes in right-sided colorectal cancer and their contribution to oncogenic processes within the tumor microenvironment. We further analyzed the biological functions enriched by the top 100 highly variable genes in the monocyte_CO2 cells, uncovering potential associations with biological processes such as leukocyte proliferation, mononuclear cell proliferation, lymphocyte proliferation, regulation of leukocyte proliferation, antigen processing and presentation, and MHC protein complex binding (Supplementary Figure S4A).

### 3.3 Temporal and communicative features of monocyte_CO2 cells

**FIGURE 3:**
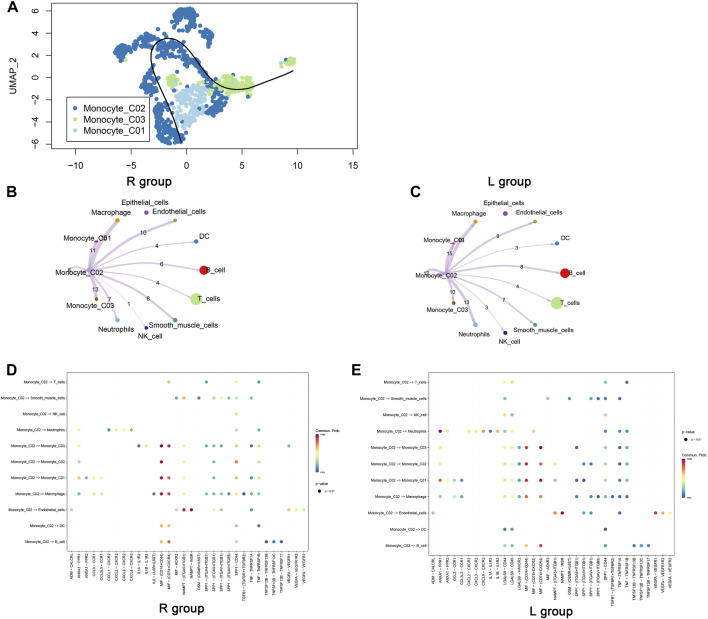
Pseudotime trajectory and intercellular communication of monocytes. **(A)** The cellular differentiation trajectory plot illustrates the differentiation pathway of monocytes, initiating from C01 and diverging toward C02 and C03. **(B, C)** The cellular communication network diagrams exhibit the communication dynamics between cells in left- and right-sided colorectal cancers originating from the Monocyte_C02 cells. **(D, E)** Dotplot diagrams display the expression of ligand–receptor pairs between monocyte_C02 cells, as the point of communication initiation, and other cells.

Figure 3A charts the evolutionary trajectory of monocyte subpopulations, transitioning from monocyte_C01 to monocyte_CO2 and monocyte_CO3 over time. Given the dynamic shifts in the tumor microenvironment, monocyte_C01 is hypothesized to represent an initial state from which they differentiate temporally. Subsequent analyses of cellular communication within the right-sided colorectal cancer (R group), in contrast to the left-sided (L group), demonstrated a marked diminution in the communication activity of monocyte_CO2 cells (Figures 3B, C). This phenomenon could reflect the biological heterogeneity between left- and right-sided colorectal cancers, potentially involving changes in cellular communication patterns and immunomodulatory processes pertinent to tumor evolution. Detailed assessments using Dotplot charts (Figures 3D, E) revealed that, against a backdrop of reduced overall cellular communication, interactions between monocyte_CO2 cells and macrophages were relatively intensified. This finding implies the formation of a more cohesive communication network among specific cell subpopulations within the tumor microenvironment, likely an adaptive response to environmental perturbations.

### 3.4 eQTL, colocalization, and directional filtering

**FIGURE 4:**
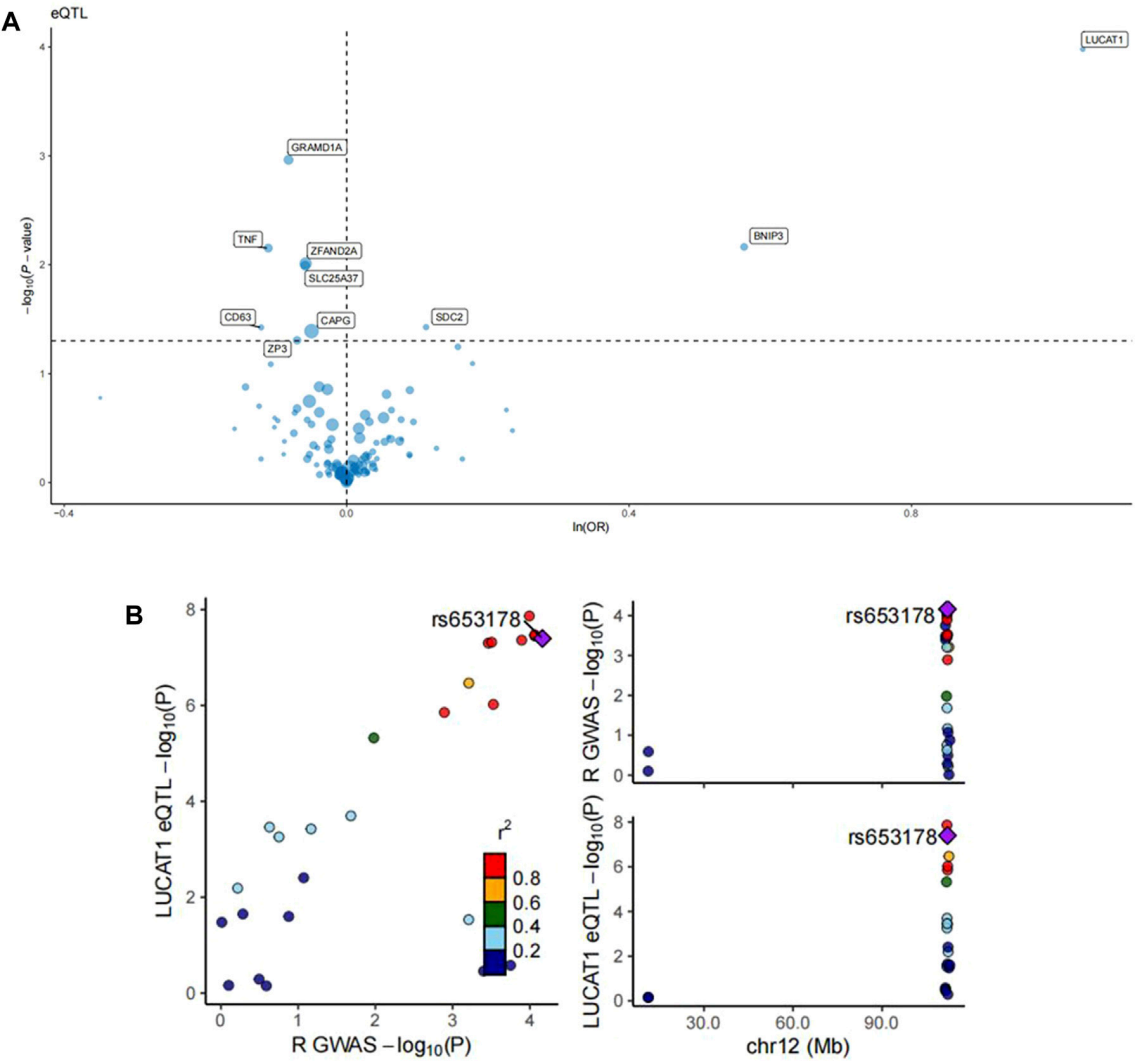
eQTL analysis of monocyte_C02 characteristic genes and regional association map of LUCAT1. **(A)** The volcano plots illustrate the Mendelian randomization (MR) results for the characteristic genes of monocyte_C02 cells in relation to colorectal cancer risk. The MR analysis utilized the Wald ratio or inverse-variance weighted method to evaluate the impact of genetic variations in the monocyte_C02 characteristic genes on colorectal cancer risk. The odds ratio (OR) for an increased risk of colorectal cancer was quantified based on per standard deviation increase in gene levels. “ln” refers to the natural logarithm, and “PVE” represents the proportion of variance explained. **(B)** The left panel depicts the relationship between the −log10(P) values of eQTLs and those of GWAS. Each point represents a single-nucleotide polymorphism (SNP), with the color indicating different r^2^ values, reflecting the degree of correlation of the SNP in both eQTL and GWAS studies. The right panel is a regional association plot for SNPs within a specific region on chromosome 12 associated with a particular phenotype. Here, the *X*-axis represents the chromosomal position, while the *Y*-axis shows the −log10(P) values of the SNP’s association with the specific phenotype. The color coding is consistent with the left panel, representing the r^2^ values.

The intersection of differential genes from monocyte_CO2 cells with those from other major cell classes and monocyte subpopulations was analyzed for Mendelian randomization with colorectal cancer. eQTL analysis identified numerous significant loci with a substantial impact on gene expression and associated colorectal cancer risk. Key gene loci, including LUCAT1, SDC2, and GRAMD1A, exhibited highly significant eQTL effects. Variations at these loci influenced gene expression substantially, with the variation at the LUCAT1 locus particularly demonstrating a robust association with increased colorectal cancer risk (Figure 4A).

Further colocalization analysis in tandem with GWAS findings pinpointed a specific SNP, rs653178, showing significant correlations in both eQTL analysis for the LUCAT1 gene and GWAS for colorectal cancer risk. The right-sided Manhattan plot reinforced the significant association signal of this SNP with colorectal cancer risk at a specific chromosomal location (Figure 4B). Steiger directional filtering lent additional support to the association directionality between SNP rs10774624 and colorectal cancer risk, suggesting the mutation precedes the cancer’s onset (Table 1).

### 3.5 Bidirectional and two-sample mendelian randomization study results indicate the relationship between LUCAT1 and the risk of colorectal cancer

**FIGURE 5:**
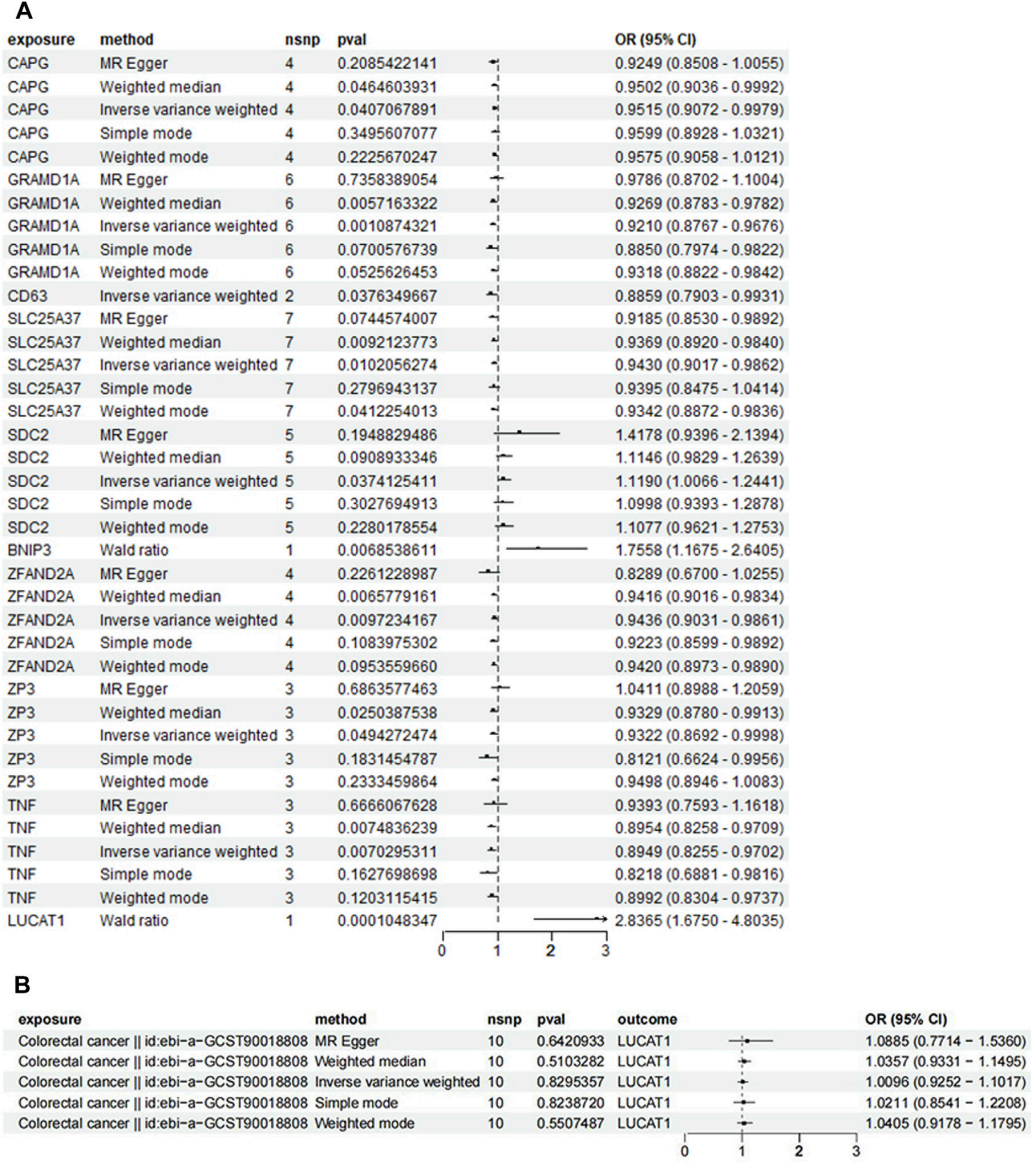
Mendelian randomization (MR) analysis of genetic variations in monocyte_C02 characteristic genes and their association with colorectal cancer risk, illustrated through forest plots. **(A)** This panel employs the Mendelian randomization approach to investigate the causal relationship between genetic variations in monocyte_C02 characteristic genes and the risk of developing colorectal cancer. Analytical methods include MR Egger, weighted median, and inverse-variance weighted approaches. **(B)** This panel utilizes MR to explore the causal relationship of genetic variations in colorectal cancer on the monocyte_C02 characteristic genes. Each point estimate represents the odds ratio (OR) associated with specific genetic variations and disease risk, accompanied by a 95% confidence interval (CI). The diagram also includes the sample size (nsnp), *p*-value (pval), and overall effect size (OR and CI) for each method. Statistical significance is typically determined by a *p*-value <0.05.

In Figure 5A, using the ebi-a-GCST90018808 dataset from the GWAS database for the forward MR analysis indicated a high positive correlation between genetic variation in LUCAT1 and the risk of developing colorectal cancer (ORwr = 2.8365 [1.6750–4.8035], *p* = 0.0001), signifying a notable elevation in cancer risk. The reverse MR analysis (Figure 5B) assessed the impact of colorectal cancer risk on the LUCAT1 gene. Employing various MR methodologies, like MR Egger and the inverse-variance weighted method, the correlation between colorectal cancer risk and the LUCAT1 gene expression was found to be non-significant, suggesting colorectal cancer is not a direct causative factor for LUCAT1 genetic variation.

**FIGURE 6:**
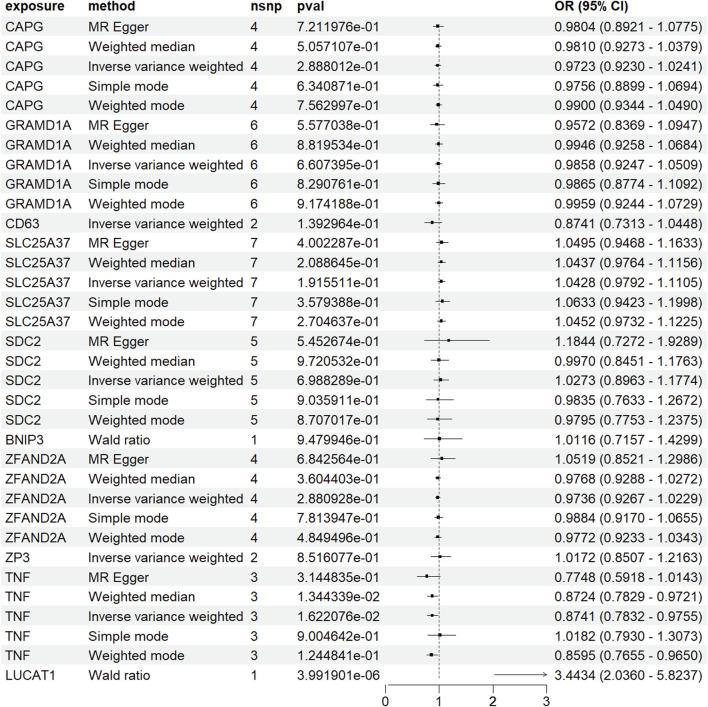
Forest plot of Mendelian randomization (MR) analysis in a validation dataset. To enhance the robustness of the MR analysis, we conducted a two-sample MR analysis using an additional dataset. Each point estimate represents the odds ratio (OR) associated with specific genetic variations and disease risk, including a 95% confidence interval (CI). The diagram also details the sample size (nsnp), *p*-value (pval), and overall effect size (OR and CI) for each method used. Statistical significance is generally determined by a *p*-value <0.05.

For validation, a two-sample MR analysis with dataset ebi-a-GCST012879 (Figure 6) corroborated the initial findings, indicating a positive correlation between increased LUCAT1 expression levels and a higher colorectal cancer risk.

### 3.6 Biological traits of LUCAT1^+^ monocytes

**FIGURE 7:**
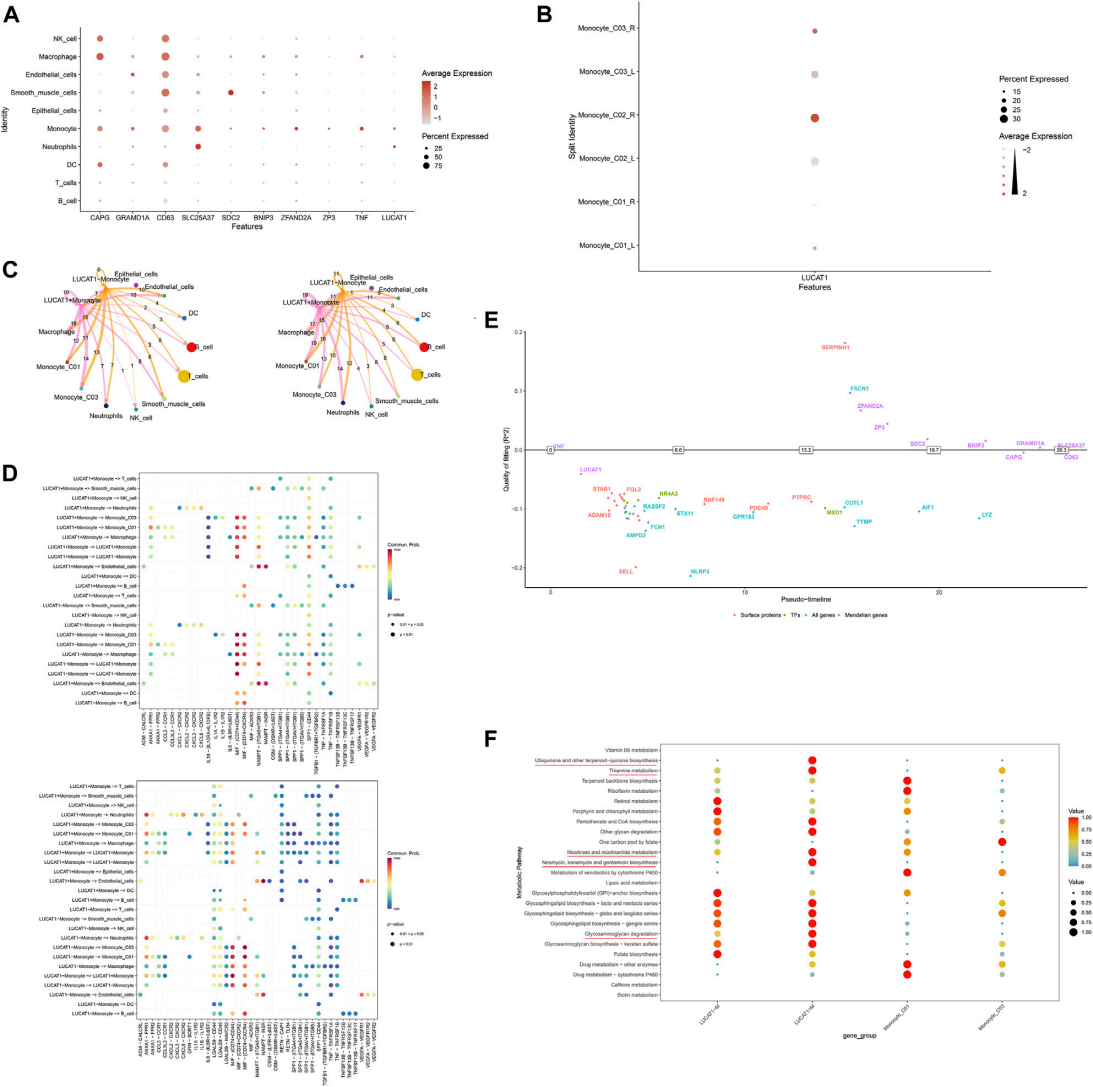
Downstream mechanisms of LUCAT1^+^ monocytes. **(A)** Expression of LUCAT1 across various cell subpopulations. Variations in size and color represent the expression level and percentage of LUCAT1 in different cell types. **(B)** LUCAT1 is highly expressed in monocyte_C02 in right-sided colorectal cancer. **(C)** Cellular communication network diagram shows the communication intensity of LUCAT1^+^ monocytes in right-sided colorectal cancer. The nodes represent different cell types, and the line width indicates the strength of communication. **(D)** Dotplot diagram displays the expression of receptor–ligand pairs involved in communication, where each dot’s size and color signify the expression level and percentage of different genes across various cell types. **(E)** The pseudo-timeline graph illustrates gene expression changes over time, with the horizontal axis representing time and the vertical axis indicating gene expression levels. **(F)** The dotplot diagram highlights the metabolic functional differences between LUCAT1^+^ and LUCAT1^−^ monocytes and other monocytes. Dots of different colors represent the activity of various metabolic pathways.

Further exploration into the role of LUCAT1 within the tumor microenvironment revealed that LUCAT1 is predominantly expressed in monocytes and neutrophils (Figure 7A) and is highly expressed in right-sided colorectal cancer (Figure 7B). Echoing previous results, LUCAT1^+^ monocytes in right-sided colorectal cancer continued to exhibit weaker communication strength (Figure 7C). Dotplot charts of LUCAT1^+^ monocytes as both communicative sources and target cells showed widespread overexpression of the receptor–ligand pair CD74 and CD44 in LUCAT1^+^ monocytes, other monocytes, and macrophages, with CD74 and CXCR4 also playing significant roles (Figure 7D). A pseudo-timeline depicted the association between gene expression and timing, with LUCAT1 primarily expressed early in the timeline (Figure 7E). Figure 7F presents metabolic function differences between LUCAT1^+^ and LUCAT1^−^ monocytes and two other monocyte groups, revealing heightened activity in metabolic functions such as glycosaminoglycan degradation, ubiquinone and other terpenoid-quinone biosynthesis, and thiamine metabolism. We further investigated the biological functions enriched by the top 100 highly variable genes in LUCAT1^+^ monocytes, revealing that these cells may be implicated in biological processes such as positive regulation of interleukin-6 production, the NF-kappa B signaling pathway, pattern recognition receptor activity, cellular response to lipopolysaccharide, and the integrin complex (Supplementary Figure S4B).

## 4 Discussion

Single-cell transcriptomic sequencing, a transformative technology widely employed in biological and medical research in recent years, enables precise analysis of gene expression patterns at the individual cell level (Wang H. et al., 2023; Xie et al., 2023). This approach is particularly vital for revealing cellular heterogeneity in disease states, especially in complex and heterogeneous cancers such as colorectal cancer. Colorectal cancer, a prevalent malignancy, is categorized into left-sided and right-sided types, each differing significantly in molecular characteristics, clinical manifestations, and prognosis (Choi and Kim, 2023; Sustic et al., 2023; Talhouni et al., 2023). For instance, left-sided colorectal cancers are commonly associated with KRAS and BRAF mutations, whereas right-sided cancers are more linked to microsatellite instability and CpG island methylation anomalies (Bond et al., 2023; Kajiwara et al., 2023). Mendelian randomization analysis, a statistical tool, utilizes these genetic variations as natural experiments to establish causative links between specific genetic markers and cancer risk, providing crucial insights into the molecular mechanisms of colorectal cancer and the development of new therapeutic strategies.

Monocytes in tumor tissues, particularly in colorectal cancer, play a complex and multifaceted role. As integral components of the immune system, they can differentiate into various cell types, including macrophages and dendritic cells, each playing intricate roles in the tumor microenvironment (Gan et al., 2023; Wu et al., 2023). On the one hand, certain differentiated monocytes, such as M2-type macrophages, may facilitate tumor growth and metastasis by secreting growth factors, pro-inflammatory cytokines, and angiogenic factors, supporting tumor proliferation and spread (Li et al., 2023; Shen et al., 2023). They may also secrete immunosuppressive molecules, aiding tumors in evading immune surveillance (Batalha et al., 2023; Gawinski et al., 2023). Conversely, some monocytes can differentiate into immunologically active cells like M1-type macrophages and certain dendritic cells, enhancing the immune response against tumor cells (Andreuzzi et al., 2022; Ito et al., 2023).

Our analysis of single-cell transcriptomic data from left- and right-sided colorectal cancers identified a higher prevalence of the monocyte_C02 subgroup in right-sided colorectal cancer. High variability genes in monocyte_C02 cells, including CXCL8, TNFAIP6, CXCL3, and SPP1, indicate a potential role in tumor promotion. Studies suggest CXCL8 can accelerate tumor cell proliferation, epithelial–mesenchymal transition (EMT), angiogenesis, and impede anti-tumor immunity (Ravi et al., 2023). CXCL3, a bioactive protein of low molecular weight, primarily recruits and activates various cells expressing the CXC chemokine receptor (CXCR)12, which is involved in cell migration, invasion, and angiogenesis and plays a crucial role in the development of cardiovascular and pulmonary diseases (Chang et al., 2023; Ji et al., 2023). Research also reveals that SPP1 impacts the tumor microenvironment by promoting inflammatory responses, immune suppression, and regulating extracellular matrix (ECM) remodeling to support tumor growth (Lv et al., 2023; Yu et al., 2023). These findings imply that the monocyte_C02 subgroup may contribute to tumor progression. The study discovered that in right-sided colorectal cancer, the reduction in communication among monocyte_CO2 cells, as well as between monocyte_CO2 cells and neutrophils, was significant. Monocytes and neutrophils can interact and influence each other’s functions within the tumor microenvironment. For instance, they can regulate each other through secreted cytokines, or in certain cases, the activation of one cell type may promote the recruitment of another cell type to the tumor microenvironment. However, this interaction is complex and may vary depending on the type of tumor, specific conditions of the microenvironment, and the host’s immune status. Monocytes can differentiate into tumor-associated macrophages (TAMs), which typically exhibit an M2 polarization state that promotes tumor growth, invasion, and metastasis by secreting growth factors, pro-inflammatory cytokines, and angiogenic factors, thus providing a favorable environment for the tumor. The reduction in communication may slow the progression of monocytes to M2-type macrophages, thereby steering right-sided colorectal cancer in a direction more favorable for immune response (Sawa-Wejksza et al., 2018). Figures 3D, E display the expression of different receptor–ligand pairs across various cell subpopulations, providing rich information on the differences in cellular communication between left- and right-sided colorectal cancers. Notably, the expression of ANXA1-FPR1 between monocytes and between monocytes and neutrophils is reduced in right-sided colorectal cancer. ANXA1 can regulate the function of T cells (especially regulatory T cells, or Tregs) by binding to FPR1, which is crucial for immune evasion in the tumor microenvironment. Specifically, the ANXA1–FPR1 complex may enhance the immunosuppressive function of Treg cells, thereby weakening the immune system’s attack on the tumor (Sawa-Wejksza et al., 2018; Tian et al., 2023). The expression of CD74-CXCR4 is decreased in right-sided colorectal cancer. In some cancers, the expression levels of CD74 and CXCR4 may influence the behavior of tumor cells, including metastasis and invasion. This may indicate a better prognosis for right-sided colorectal cancer (Bian et al., 2023). The biological functions of monocyte_CO2 cells are primarily enriched in processes such as leukocyte proliferation, mononuclear cell proliferation, lymphocyte proliferation, regulation of leukocyte proliferation, antigen processing and presentation, and MHC protein complex binding. Monocytes, including monocytes and certain types of lymphocytes, can further differentiate into macrophages and dendritic cells, which play pivotal roles in tumor immune responses. Macrophages can have either tumor-promoting or tumor-suppressing effects depending on their states within the tumor microenvironment. The proliferation and activation of monocytes are crucial in the tumor immune editing process, as they influence immune responses through cytokine production and antigen presentation. The coordinated action of these immune functions is essential for eliciting effective tumor-specific immune responses. The coordination of immune functions such as MHC protein complex binding, MHC class II protein complex binding, and antigen processing and presentation is crucial for triggering effective tumor-specific immune responses. The ability of immune cells to efficiently recognize and respond to tumor cells largely depends on the effective processing and presentation of tumor antigens, as well as the correct expression and function of MHC molecules. This suggests that the increase in monocyte_CO2 cells is likely related to the immune environment of right-sided colorectal cancer. The reason for this could be that right-sided colorectal cancers often have a higher microsatellite instability (MSI) and BRAF mutation rate, while left-sided cancers are more associated with chromosomal instability pathways. This could also explain why right-sided colorectal cancers tend to have higher immune response rates.

Our two-sample Mendelian randomization and reverse Mendelian randomization analyses identified genetic variations in LUCAT1 as risk factors for colorectal cancer, supported by directional filtering and colocalization analyses. The mutation at the rs10774624 locus was deemed causally related to colorectal cancer onset, and additional colocalization analysis revealed another potential disease risk-related locus sharing genetic variation, rs653178. Although research on these loci is scarce, known biological functions of LUCAT1 and eQTL results suggest a strong promotive effect of its SNPs on colorectal cancer development.

To further explore the potential role of LUCAT1, we conducted separate analyses of the functions of LUCAT1^+^ and LUCAT1^−^ monocytes in tumor tissues. LUCAT1 expression was found to be higher in right-sided colorectal cancer, supporting the hypothesis that LUCAT1 is a risk factor that may contribute to the development of right-sided colorectal cancer. Generally, LUCAT1^+^ monocytes demonstrated weaker communication strength than LUCAT1^−^ monocytes, which could be attributed to the typically better prognosis of right-sided colorectal cancer, leading to lower levels of malignancy cell communication. Moreover, monocyte_C02 cells showed the most robust communication with other monocytes, likely linked to monocyte differentiation processes, especially involving MIF interactions with CD74 and CD44. In tumorigenesis, MIF-CD74 interactions are known to promote tumor cell growth and survival, with the expression levels of MIF being closely tied to tumor aggressiveness and prognosis in certain types of cancer. MIF-CD44 interactions are also crucial in affecting cell–ECM interactions, particularly important in the migration and metastasis of tumor cells. The pronounced activity of LUCAT1 in the early stages of monocyte differentiation may be associated with its role in facilitating malignant differentiation and the onset of tumors. An analysis of differential metabolic pathways revealed increased activity in glycosaminoglycan degradation in LUCAT1^+^ monocytes. Glycosaminoglycans (GAGs), crucial biomolecules containing uronic acid and amino sugar residues, disrupt cell–cell adhesion during tumor cell dissociation and invasion. The modification of cadherin (E-cadherin) by β1,6 N-acetylglucosaminyltransferase V (GnT-V), which adds β1,6-N-acetylglucosamine (β1,6GlcNAc)-branched N-glycans, impairs cell adhesion and aids in tumor cell invasion. Additionally, the α2,6-sialylation terminal structure interferes with tumor cell adhesion (Ahn et al., 2009; Shen et al., 2022). The biological functions of LUCAT1^+^ monocytes are markedly enriched in pathways, including positive regulation of interleukin-6 production, NF-kappa B signaling, pattern recognition receptor activity, cellular response to lipopolysaccharide, and integrin complexes. Among these, IL-6 is a multifunctional cytokine that plays a pivotal role in the initiation and progression of tumors. Elevated levels of IL-6 are associated with various types of cancers, contributing to the proliferation, survival, and migration of tumor cells. IL-6 facilitates inflammatory reactions and the formation of the tumor microenvironment by activating the JAK/STAT3 signaling pathway, which aids in tumor growth and immune evasion (Johnson et al., 2018). The NF-κB signaling pathway is crucial in the regulation of inflammatory and immune responses. This pathway is often activated in cancer, enhancing tumor cell proliferation, survival, and invasion while inhibiting apoptosis. Activation of NF-κB is also intimately connected with the recruitment of inflammatory cells within the tumor microenvironment and the generation of tumor-promoting inflammation (Lee et al., 2007). Pattern recognition receptors (PRRs) play an essential role in innate immunity by recognizing pathogen-associated molecular patterns (PAMPs) and damage-associated molecular patterns (DAMPs). Within the tumor microenvironment, activation of PRRs can promote inflammatory responses that may favor the proliferation and survival of tumor cells. Integrins, as receptors on the cell surface, mediate interactions between cells and the extracellular matrix (ECM) that are crucial for cell migration, proliferation, and survival (Amarante-Mendes et al., 2018). In cancer, integrin activation can enhance the invasiveness and metastatic potential of tumor cells, remodeling the tumor microenvironment through interactions with the ECM. This further suggests that the expression of LUCAT1 may facilitate the progression of tumors.

## 5 Conclusion

Through single-cell transcriptomic sequencing, we discovered that the monocyte_C02 subpopulation is more prevalent in right-sided colorectal cancer, and its highly expressed genes, such as CXCL8, TNFAIP6, CXCL3, and SPP1, may be implicated in tumor growth promotion. Notably, we observed that the expression of LUCAT1 in these monocytes could be associated with the occurrence of colorectal cancer, with Mendelian randomization analysis further indicating a direct link between genetic variations in LUCAT1 and colorectal cancer risk. Additionally, LUCAT1^+^ monocytes were found to be more active in right-sided colorectal cancer, potentially influencing the glycosaminoglycan degradation pathway, thereby disrupting cell adhesion and facilitating tumor cell invasion. In summary, LUCAT1 is not only a risk factor for the development of colorectal cancer but may also participate in the progression of the disease by modulating the functions of monocytes and the interactions within the tumor microenvironment.

This provides a new perspective in uncovering and understanding the complex biological mechanisms of right-sided colorectal cancer and offers valuable insights for developing potential therapeutic strategies targeting LUCAT1. Future advancements in these findings may contribute to promoting more personalized cancer treatment approaches, particularly tailored therapies for different subtypes of left- and right-sided colorectal cancer, ultimately improving patient prognosis.

## Data Availability

The original contributions presented in the study are included in the article/Supplementary Material, further inquiries can be directed to the corresponding authors.
