# Supplementary material for: Single-cell transcriptomics and Mendelian randomization reveal LUCAT1’s role in right-sided colorectal cancer risk
Source: Front Genet. 2024 Apr 22;15:1357704. doi: 10.3389/fgene.2024.1357704 (PMC11070547; doi:10.3389/fgene.2024.1357704)

Supplementary Material

## Supplementary Figures

**Supplementary Figure 1.** Quality control and batch effect removal in single-cell transcriptome sequencing. (A–B) Violin plots for scRNA-seq data quality control (A) and after quality control (B) measures by sample. Plots show the number of genes (nFeature_RNA), the number of UMIs (nCount_RNA), the percent of mitochondrial content (percent.mt), and the percent of ribosome content (percent.rb) for samples. (C–D) The illustration presents key aspects of single-cell transcriptomic data variability and batch-effect correction. Figure C exhibits a Principal Component Analysis (PCA) scatter plot (C) representing the cellular distribution prior to batch correction, with cells color-coded based on their original identities (orig.ident). Each dot symbolizes an individual cell's transcriptome complexity, as delineated by PC_1 and PC_2. Figure D displays a Harmony-adjusted PCA scatter plot (D) post batch-effect alignment, highlighting the repositioning of cells to mitigate technical variations across different identities. (E) Standard Deviation Analysis in Harmony Dimensions. This plot displays the standard deviation for each Harmony dimension, highlighting the decrease in variability with higher dimensions. This suggests effective batch correction in single-cell RNA sequencing analysis.


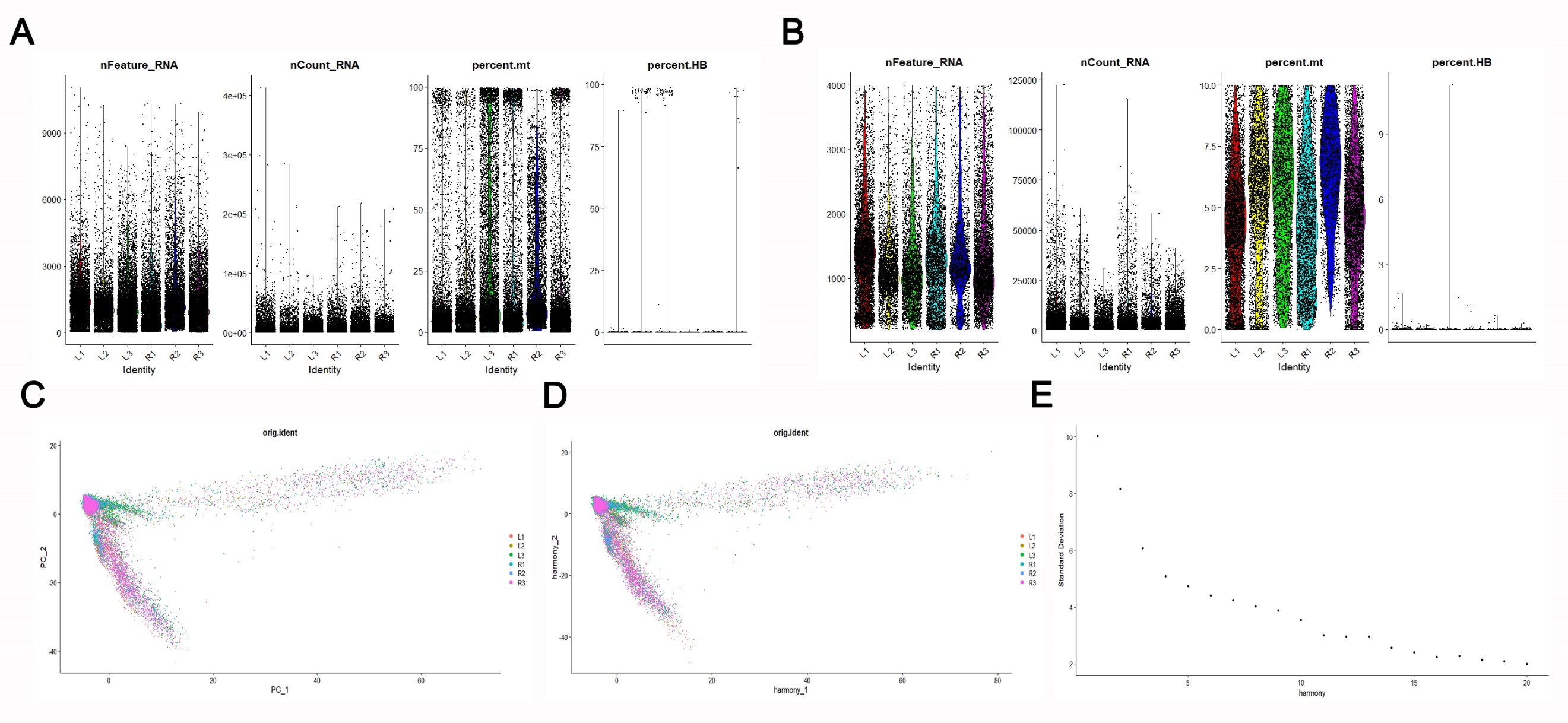


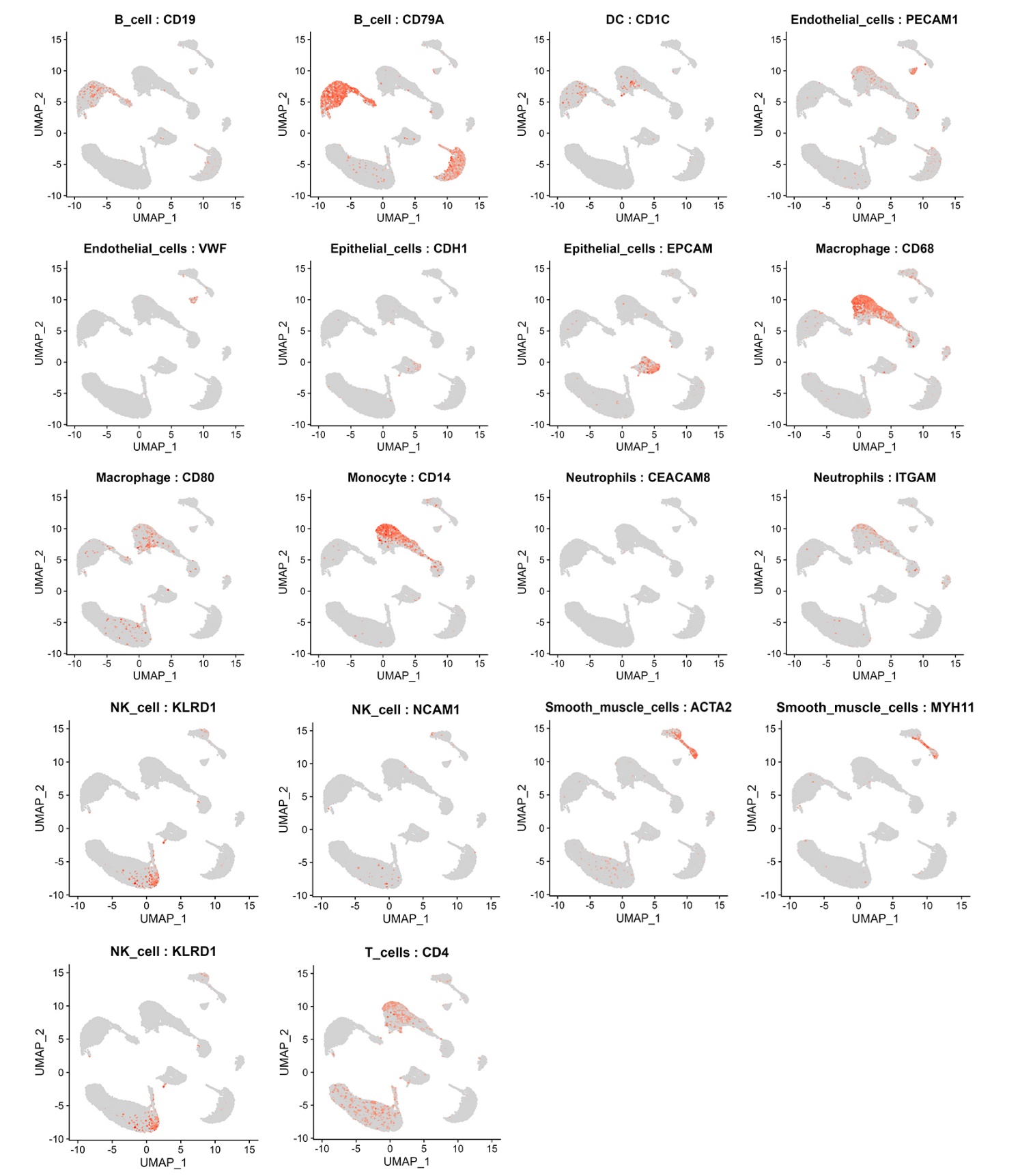
**Supplementary Figure 2**. Markers of the cellular subpopulations

**Supplementary Figure
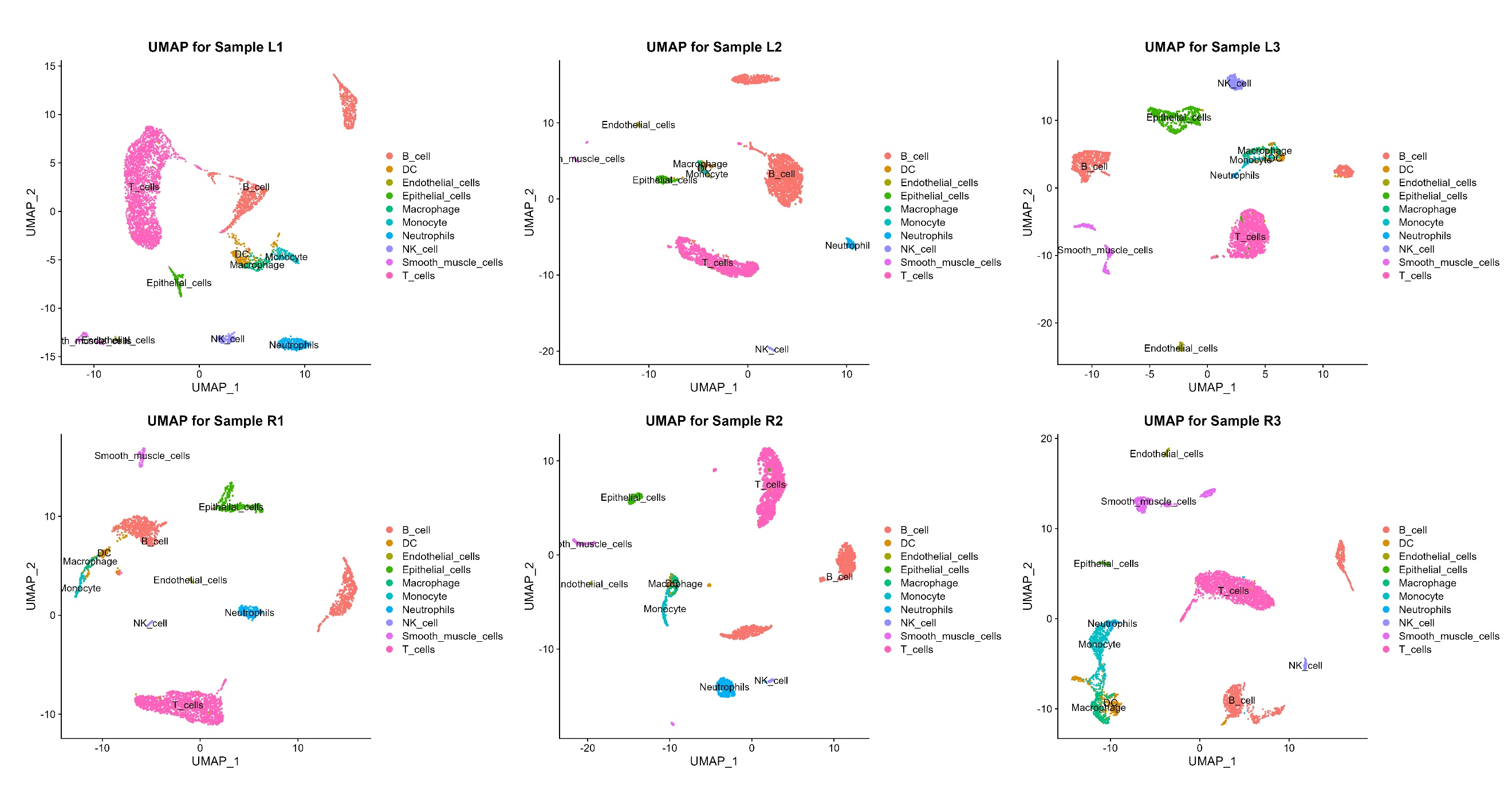
3**. Dimensionality reduction clustering diagrams (UMAP) for each of the six samples.

**Supplementary Figure 4**. Functional enrichment analysis of highly variable genes in cellular subpopulations. (A) GO and KEGG enrichment of the top 100 highly variable genes in Monocyte_CO2. (B) GO and KEGG enrichment of the top 100 highly variable genes in LUCAT1^+^ monocytes.


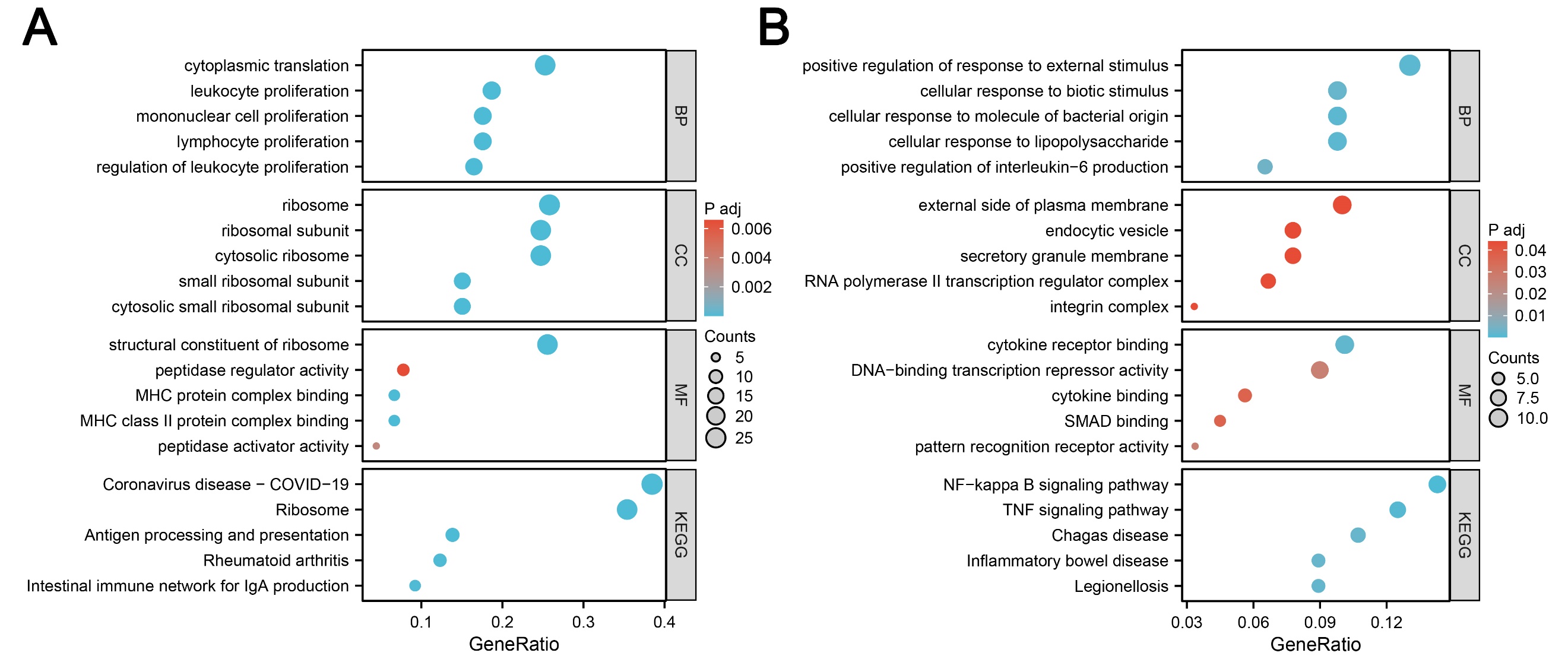

Supplement: Supplementary file 1 [file Table1.DOCX]
